# Aggressive CD5-Positive Primary Bone Marrow Diffuse Large B-Cell Lymphoma with Leukemic Presentation

**DOI:** 10.1155/2021/2628100

**Published:** 2021-10-14

**Authors:** Mark G. Evans, Sherif A. Rezk, Lauren C. Pinter-Brown, Xiaohui Zhao

**Affiliations:** ^1^Department of Hematopathology, Division of Pathology and Laboratory Medicine, The University of Texas MD Anderson Cancer Center, Houston, TX, USA; ^2^Department of Pathology and Laboratory Medicine, University of California Irvine (UCI) Medical Center, Orange, CA, USA; ^3^Department of Medicine, Division of Hematology/Oncology, University of California Irvine (UCI) Medical Center, Orange, CA, USA

## Abstract

Primary bone marrow diffuse large B-cell lymphoma is an exceedingly rare form of non-Hodgkin lymphoma. It may demonstrate a leukemic presentation, and a proportion of cases have CD5 expression. The prognostic implications of this CD5-positivity remain unknown. Here, we present a 78-year-old man who presented with circulating peripheral blood lymphoma cells and a hypercellular marrow involved by diffuse large B-cell lymphoma, germinal center B-cell subtype. The patient responded favorably to six cycles of etoposide, prednisone, vincristine, cyclophosphamide, doxorubicin, and rituximab (EPOCH-R) and intrathecal methotrexate. He unfortunately relapsed in several enlarged inguinal lymph nodes and succumbed to the lymphoma approximately one year after diagnosis, demonstrating the particularly aggressive clinical course of his disease.

## 1. Introduction

Primary bone marrow lymphoma (PBML) accounts for only 1.16% of all lymphomas and most frequently presents as diffuse large B-cell lymphoma (DLBCL) [1]. PBML was initially described as bone marrow involvement at first presentation [1]. However, some authors have noted that initial bone marrow disease can also occur as a secondary manifestation of nodal lymphoma and that these cases should not be considered true PBML [2]. Alternatively, PBML has been defined as involving only the bone marrow [3] or without any lymphadenopathy [4]. Subsequently, additional PBML subtypes including small-cell B-cell lymphomas, and not DLBCL, have been observed [5].

Given the rarity of this disease, reliable epidemiologic information is not available. Only case reports and small case series have been published, which typically associate PBML with a poor prognosis [6]. Although one study described a cohort of patients who achieved potential cures [1], our case followed the aggressive course typically associated with such cases, where our patient quickly expired following relapsed disease and intensive chemotherapy.

## 2. Case Report

**Figure 1:**
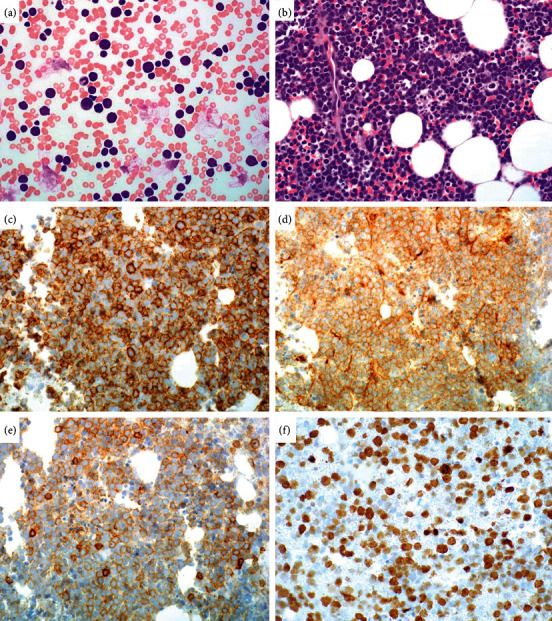
The patient's peripheral blood features medium and large-sized cells with scant cytoplasm and irregular nuclear contours (a; 400x magnification). Approximately 70–80% of the cellular elements within his hypercellular bone marrow biopsy show morphologic characteristics similar to those seen in the peripheral blood (b; 400x magnification). The cells are positive for CD20 (c), CD10 (d), and CD5 (e) by immunohistochemistry (400x magnification). Ki-67 demonstrates a high proliferation index of 80% (f; 400x magnification).

A 78-year-old man presented with a one-week history of anorexia and generalized weakness. His medical history was significant for metastatic papillary thyroid cancer, treated with radioactive iodine ablation, thyroidectomy, and axitinib. Computed tomography (CT) imaging demonstrated interval increase in splenic and hepatic size, but no concerning masses or lymphadenopathy. However, the patient's serum lactate dehydrogenase (LDH) was 4866 U/L, and his white blood cell count was 454 × 10^3^ /*μ*L. His peripheral blood smear was notable for medium and large-sized lymphocytes with irregular nuclear contours accounting for 15% of circulating white blood cells (Figure 1(a)).

A subsequent bone marrow biopsy was hypercellular with 70–80% involvement by sheets of large cells demonstrating blast-like morphology with scant cytoplasm, irregular nuclear contours, and prominent nucleoli. Numerous apoptotic bodies and mitotic figures were identified. By immunohistochemistry, the malignant cells were positive for CD20, PAX-5, CD5, CD10, MUM1, and BCL-2, while negative for EBV, CD30, BCL-6, cyclin D1, SOX-11, TdT, and MYC (Figures 1(b)–1(e)). A high Ki-67 proliferative index of 80–90% was observed. The bone marrow karyotype was reported as 47, XY, +7, del(13) (q12q22), add(14) (q32.3) (8)/46, and XY(12). Concurrent fluorescence in situ hybridization (FISH) was negative for BCR/ABL1, t(14; 18), IGH, BCL-6, and MYC rearrangements. Overall, these findings were consistent with CD5-positive diffuse large B-cell lymphoma, germinal center B-cell (GCB) subtype.

The patient received six cycles of etoposide, prednisone, vincristine, cyclophosphamide, doxorubicin, and rituximab (EPOCH-R), with intrathecal methotrexate and 50% dose reduction of doxorubicin and vincristine. Repeat bone marrow biopsy following the second cycle demonstrated a normocellular marrow with no lymphoma involvement, and follow-up imaging confirmed a complete response. Five months later, the patient developed relapse in several right inguinal lymph nodes, which demonstrated morphologic and immunohistochemical features identical to his initial bone marrow biopsy. He received six cycles of gemcitabine and oxaliplatin plus rituximab and intrathecal methotrexate, with radiologic evidence of a complete response after the third cycle. Unfortunately, the patient relapsed in the form of an enlarging abdominal mass and shortly thereafter expired, approximately one year after initial diagnosis.

## 3. Discussion

Primary bone marrow lymphoma is a rare lymphoma that usually presents as DLBCL. Our patient demonstrated relapse and an aggressive clinical course following leukemic presentation of primary bone marrow DLBCL. He was previously diagnosed with thyroid cancer and received axitinib. The published literature has yet to discuss a possible correlation between PBML and previous malignancies or their treatments. One case series has described a small cohort of patients with PBML, DLBCL subtype who demonstrated a better-than-expected prognosis [1], where each of the individuals presented with good performance status, favorable international prognostic index (IPI), and had received rituximab-based therapy. In most studies, the overall survival is dismal. While no recommended therapy exists for PBML, most studies document the use of rituximab, cyclophosphamide, doxorubicin, vincristine, and prednisone (R-CHOP) and a median survival of 9.6 months [7]. A review of 75 patients within the medical literature reported only 15 (20%) surviving, with follow-up times typically less than 24 months [1].

CD5-positivity is a relatively rare occurrence in DLBCL, observed in up to only 10% of cases [8]. Of interest, CD5 expression was demonstrated in both the patient's bone marrow biopsy and subsequent inguinal lymph node biopsy at relapse, with both specimens showing a CD10-positive GCB subtype. While the observed MUM1 expression is uncommon in GCB cases, it has been reported in 13% of 601 DLBCL patients in one study [9]. An alternate diagnosis of intravascular large B-cell lymphoma (IVLBCL) might be entertained, as it features frequent MUM1-positivity, bone marrow involvement, and CD5 expression in up to 38% of cases [10]. However, IVLBCL displays CD10-positivity in only 13% of cases and rarely occurs in lymph nodes [11]. Ultimately, the patient's findings favored the presence of a CD5-positive DLBCL, GCB subtype.

Yamaguchi et al. published a large series of 109 patients with DLBCL and CD5 expression [12]. The authors noted that these cases were more likely to present at the advanced stage, greater than 60 years of age, with elevated serum LDH and B symptoms compared to those without CD5-positivity. Overall, only 16 of the patients (14.7%) could be diagnosed as PBML. Nonetheless, the authors stated that bone marrow was the most common extranodal site of CD5-positive DLBCL. While CD5-positivity has been described in a large proportion of primary bone marrow DLBCL, CD5-negative cases have demonstrated similar features and outcomes [13, 14]. Whether CD5 expression influences outcome in PBML remains uncertain, although the devastating leukemic presentation and eventual relapse in our patient provide additional evidence for the poor prognostic significance of CD5.
